# Book Reviews

**Published:** 1898-05

**Authors:** 


### Book Reviews.

ANNUAL AND ANALYTICAL CYCLOPÆDIA OF PRACTICAL MEDICINE. By Charles E. de M. Sajous, M. D., and one hundred associate editors, assisted by corresponding editors and correspondents. Illustrated with chromo-lithographs and maps. Volume I. Philadelphia, New York, Chicago. The F. A. Davis Company Publishers, 1898.

In this age of great advancement in the sciences, it is remarkable what a mass of literature can be collected upon any given subject if the articles in the leading medical journals of the world are examined for this purpose. The average practitioner is too busy to read all that may be written, but he should be expected to know the results of all that is done in his profession. This book does for the practitioner the same service as though he should call upon a consultant to tell him in each case. Inasmuch as the country practitioner is far removed from the leading centers of medical education and thought, and as the physicians who need consultations at times would prefer to have their own authority in the books, this Annual and Cyclopædia is a great help and a treasure. Some months ago THE SOUTHERN MEDICAL RECORD published a letter from Dr. Sajous to Dr. Gaston, one of the associate editors, asking for his commentation of the article on Aneurisms. In this letter the scope of the work was well defined, so that the readers of the RECORD are conversant with the change from the Annual to the above caption. The second volume will be issued shortly, beginning with the diseases following Bright's Disease in alphabetical order.

The subject of Empyema is to appear in this second volume and will be signed by J. McF. Gaston and J. McF. Gaston, Jr., who, we are informed, have just finished its preparation.

The work is respectfully dedicated to the American medical profession as an expression of devotion by the editor, and we are certain that the profession will accredit to Dr. Sajous the devotion which he professes, since as editor of the "Annual of the Universal Medical Sciences" he has done more than any other one man to preserve the literature of the medical fraternity.

The sections of this new work which have been prepared *in toto* by associates for this volume are those on "Addison's Disease," "Angina Pectoris," "Astigmatism," "Actinomycosis," "Anthrax," "Acetonuria," "Albuminuria," "Alcohol," "Antipyrine," "Atropine," "Belladonna," "Blepharitis," and "Bright's Disease."

The classification adopted is, to a certain degree, a novel one, general subjects alone appearing in the lists.

The whole work will comprise six volumes of much larger size than the Annual heretofore published, the first one containing six hundred pages of six hundred words or more.

The cost of this by subscription is thirty dollars, and, as the case with the Annual, the sets are not broken. We wish the new work the great success which we are confident it will receive.

